# Decrease in number of mast cells in resected nasal polyps as an indicator for postoperative recurrence of chronic rhinosinusitis

**DOI:** 10.1002/iid3.261

**Published:** 2019-06-17

**Authors:** Yuichi Teranishi, Denan Jin, Sakurako Takano, Kishiko Sunami, Shinji Takai

**Affiliations:** ^1^ Department of Otolaryngology, Head and Neck Surgery, Osaka City University Graduate School of Medicine Osaka City University Osaka‐City Osaka Japan; ^2^ Department of Innovative Medicine, Graduate School of Medicine Osaka Medical College Takatsuki Osaka Japan

**Keywords:** chymase, mast cells, nasal polyps, rhinosinusitis, tryptase

## Abstract

**Background:**

In the clinical setting, chronic rhinosinusitis with nasal polyps (CRSwNP) is usually divided into eosinophilic‐CRS (ECRS) and non‐ECRS (NECRS) in Japan. Patients with the former are believed to be at risk for postoperative recurrence of CRS. However, some patients have been missed according to these phenotypic classifications due to the low number of infiltrating eosinophils in polyp tissues.

**Objective:**

In the present study, we attempted to identify cellular or molecular candidate markers to predict nasal polyp recurrence.

**Methods:**

Nasal polyps were collected from 32 patients with CRSwNP who had undergone an endoscopic sinus surgery. These patients were divided into ECRS and NECRS groups in accordance with the Japanese Epidemiological Survey of Refractory Eosinophilic Chronic Rhinosinusitis (JESREC) scoring system and the number of eosinophils in polyp tissues. Unclassifiable patients were referred to as the unknown group.

**Results:**

Eosinophil infiltration in resected nasal polyps was most evident in the ECRS group. However, the number of mast cells and tryptase‐positive cells in nasal polyps were significantly lower in ECRS and unknown groups compared with the NECRS group. A significant positive correlation was detected between the JESREC score and number of eosinophils. The numbers of mast cells and tryptase‐positive cells were negatively correlated with the JESREC score in all included samples. Significant positive correlations were detected between the number of transforming growth factor β1‐positive cells and the number of mast cells, tryptase‐positive cells, and chymase‐positive cells mast cells.

**Conclusions and clinical relevance:**

These findings indicated that the enumeration of mast cells in resected polyps may be another approach to predict postoperative polyp recurrence in CRSwNP patients.

## 1 INTRODUCTION

Rhinosinusitis is an inflammation or swelling of nasal mucous membranes and usually starts with cold‐like symptoms such as a runny, stuffy nose, and facial pain. Conditions that persist for less than 4 weeks or more than 12 weeks are defined as acute rhinosinusitis or chronic rhinosinusitis (CRS), respectively.^1^ CRS can be divided into two types in accordance with polyp formation: chronic rhinosinusitis with nasal polyps (CRSwNP) and CRS without nasal polyps (CRSsNP). Compared with CRSsNP, CRSwNP is characterized by more severe inflammatory mucosal reactions coincident with fibrosis, goblet cell hyperplasia, and basement membrane thickening and occasionally accompanying anosmia or dysgeusia.^2^ Although pharmacological intervention, such as topical or oral steroid administration, can decrease sinus mucosa inflammation and reduce polyp size,^3^ otolaryngologists recently prefer to select endoscopic sinus surgery (ESS) to remove polyps in CRSwNP patients.^4^ This surgical resection is very effective for most people and provides rapid symptom relief. Unfortunately, recurrence of nasal polyps will happen in some postoperative patients, especially in patients with ethmoid‐dominant shadow evaluated by computed tomography (CT) or peripheral blood eosinophil ratio ≥5%.^5^ Moreover, histological examinations performed on these resected polyps indicate that there is a strong positive correlation between peripheral blood eosinophils and infiltrated eosinophils in nasal polyps.^3^ Since eosinophilic CRS patients with polyps have a strong tendency to recur after ESS, the Oto‐Rhino‐Laryngological Society of Japan designated a new phenotype of CRSwNP called eosinophilic‐CRS (ECRS) to distinguish it from other CRS [non‐ECRS (NECRS)].^6^ In addition, a detailed scoring system and algorithm for diagnosis of ECRS were created by the Japanese Epidemiological Survey of Refractory Eosinophilic Chronic Rhinosinusitis (JESREC) Study.^5^ In this study, the following indices were used: bilateral disease sites, presence of nasal polyps, ethmoid sinus‐dominant disease in CT scans, and peripheral blood eosinophilia. The total maximum score evaluated by these indices is 17 points. If the calculated JESREC score is ≥11 points in a preoperative patient, then the case is temporarily diagnosed as ECRS. Sensitivity and specificity of this criterion were 83% and 66%, respectively.^5^ The number of eosinophils in the resected polyp (including biopsy) cross section is also an important index. Criteria for definitive diagnosis of ECRS are >70 eosinophils per high‐power field (HPF, 400×) in the cross‐section and JESREC score ≥11 points.^5^ According to the comorbidity of bronchial asthma or presence of aspirin intolerance or nonsteroidal anti‐inflammatory drugs (NSAIDs) intolerance, ECRS patients can also be divided into mild or severe ECRS.^5^ In view of the strong tendency for ECRS patients to recur after ESS, the Ministry of Health, Labour, and Welfare of Japan has classified ECRS as a designated incurable disease to better manage and support these postoperative patients. Presently, in Japan, postoperative maintenance therapies are good, and the medical treatment costs covered by insurance is higher in ECRS patients than others. Therefore, the definitive diagnosis for ECRS is often cautiously made by otolaryngologists. However, despite having high JESREC scores, some patients are misdiagnosed with NECRS due to the low number of infiltrating eosinophils in polyp tissues.

To resolve this issue, cellular and molecular distribution patterns in surgically resected nasal polyps from CRSwNP patients, such as mast cells,^7^ transforming growth factor β1 (TGF‐β1),^8^ and periostin,^9^ which are suspected to be important in the formation of nasal polyps under CRS, were histologically examined. Among them, we found that the number of mast cells in resected polyps was significantly lower in patients who tended to have postoperative polyp recurrence. Moreover, by analyzing all recruited subjects, a strong negative correlation between the number of mast cells and JESREC score was calculated. It is well known that high JESREC score in CRSwNP patients indicates risk of postoperative polyp recurrence. Since toluidine blue staining is as simple to perform as hematoxylin‐eosin (HE) staining, we feel that mast cell estimation in resected polyps may become another approach to predict postoperative polyp recurrence in CRSwNP patients.

## 2 METHODS

### 2.1 Polyp collection

Nasal polyps were collected from 32 patients with CRSwNP who had undergone ESS from April 2016 to October 2018 at the Department of Otolaryngology, Head and Neck Surgery, Osaka City University Hospital.

Before surgery, informed consent was obtained from all patients. This experimental protocol was approved by the Human Studies Committee of the Graduate School of Medicine and Faculty of Medicine, Osaka City University (authorization number: 3606).

### 2.2 General histological and immunohistological studies

Resected polyps were fixed in Carnoy's solution (Muto Pure Chemicals Co., Ltd. Tokyo, Japan) overnight and displaced with 100% ethanol to prepare paraffin blocks. For histological staining, 4‐μm serial cross sections were prepared. To identify the number of eosinophils and mast cells, HE staining and toluidine blue staining were used, respectively. Azan Mallory staining was also used to determine collagen distribution in polyps. The first and second serial cross sections were selected to observe the distribution of eosinophils (HE staining) and collagen fibers (Azan Mallory staining) on polyp cross sections using standard staining protocols. The fourth cross‐section was used to identify distribution and number of mast cells. In brief, deparaffinized sections were immersed in 0.5% toluidine blue solution (pH 4.8) for about 15 minutes, fractionated with 0.5% glacial acetic acid solution, and mounted after drying.

The third, fifth, and sixth serial cross‐sections were used to identify distribution of TGF‐β1, tryptase, and chymase by using anti‐TGF‐β1 antibody (ARP37894‐P505, 1:100 dilution; Aviva Systems Biology, San Diego, CA), anti‐tryptase antibody (M7052, 1:800 dilution; Dako, Glostrup, Denmark), and anti‐chymase antibody (mouse monoclonal antibody against human mast cell chymase, 2D11G10D, 1:1,000 dilution; a kind gift from Suzuki, Katakura Industries Co., Saitama, Japan), respectively. Both tryptase and chymase are secretory granule‐derived proteinases contained in mast cells. Human mast cells are usually classified into two types: mast cells that contain both tryptase and chymase (MC_TC_ cells) and mast cells that contain tryptase only (MC_T_ cells).^10^ Thus, tryptase is usually considered as a protein marker of mast cells and the total number of tryptase‐positive cells reflects the sum of the two mast cell subtypes.

Since a typical mast cell has a mean diameter of 8 to 15 μm, these respective mast cell subtypes may be more accurately examined when using serial sections to stain mast cells, tryptase‐positive cells, and chymase‐positive cells. For example, if we cut two serial cross sections at the 4‐μm thickness, then the majority of mast cells may be simultaneously found in both sections. Through the detection of both tryptase‐ and chymase‐positive cells, the ratio of MC_TC_ and MC_T_ can also be calculated. The number of tryptase‐positive cells indicates the total number of mast cells, while the number of chymase‐positive cells, indicates the number of MC_TC_ cells. In the present study, the ratio of MC_TC_ cells was expressed as a percentage of MC_TC_ cells among tryptase‐positive cells. Mast cells also express TGF‐β1,^11^ therefore, we performed TGF‐β1 staining along with toluidine blue staining.

The seventh, eighth, ninth, and tenth serial cross‐sections were used to identify the distribution of proliferating cell nuclear antigen (PCNA)‐positive cells (1:100 dilution; Dako), vimentin‐positive cells (1:70 dilution; Dako), alpha‐smooth muscle actin (α‐SMA)‐positive cells (1:200 dilution; Dako), and periostin (sc‐49480, 1:100 dilution; Santa Cruz Biotechnology, Santa Cruz, CA).

Immunohistological staining using the abovementioned antibodies was performed in accordance with protocols described elsewhere.^12^ In brief, deparaffinized sections were incubated with respective antibodies overnight at 4°C, followed by reaction with components from a labeled streptavidin‐biotin peroxidase kit (Dako LSAB kit; Dako, Carpinteria, CA) that included 3‐amino‐9‐ethylcarbazole for color development.

In the present study, the cellular number in each cross section was counted at HPF (400×) in the three densest areas, and the mean value was used for statistical analysis.

### 2.3 Grouping

To classify ECRS and NECRS phenotypes among recruited CRSwNP patients, the number of eosinophils was evaluated on HE‐stained cross sections of nasal polyps from patients undergoing ESS. Final diagnosis was made in accordance with the JESREC scoring system as follows^5^: (a) disease sides (bilateral = 3, unilateral = 0); (b) nasal polyps (presence = 2, absence = 0); (c) CT shadow (ethmoid ≥ maxillary = 2, ethmoid ≤ maxillary = 0); and (d) eosinophils in peripheral blood (2%–5% = 4, 5%–10% = 8, 10% ≤ 10). Patients with a JESREC score ≥ 11 and a polyp tissue eosinophil count ≥70 were diagnosed with ECRS, while patients with a JESREC score ≤10 and a polyp tissue eosinophil count <70 were diagnosed with NECRS. However, despite the high scores obtained from the JESREC scoring system, we could not differentiate some patients into either group due to the low number of eosinophils in polyp tissues (<70) evaluated by HE‐stained sections. These patients were referred to as the unknown group in the present study.

### 2.4 Statistical analysis

All numerical data are expressed as the mean ± SEM. Significant differences among mean values of multiple groups were evaluated using one‐way analysis of variance followed by post hoc analysis (Fisher's test). Pearson's correlation coefficient was measured to test the linear relationship between two variables using linear regression analysis. *P* < .05 was considered statistically significant.

## 3 RESULTS

Thirty‐two CRSwNP patients were enrolled in the present study. When we evaluated these patients by using the JESREC scoring system, 17 and 8 patients met the criteria for ECRS and NECRS, respectively. However, the remaining seven patients were unclassifiable because of the low number of eosinophils in polyp tissues (<70) on HE‐stained sections. Therefore, we classified these seven patients as the “unknown group.”

**Figure 1:**
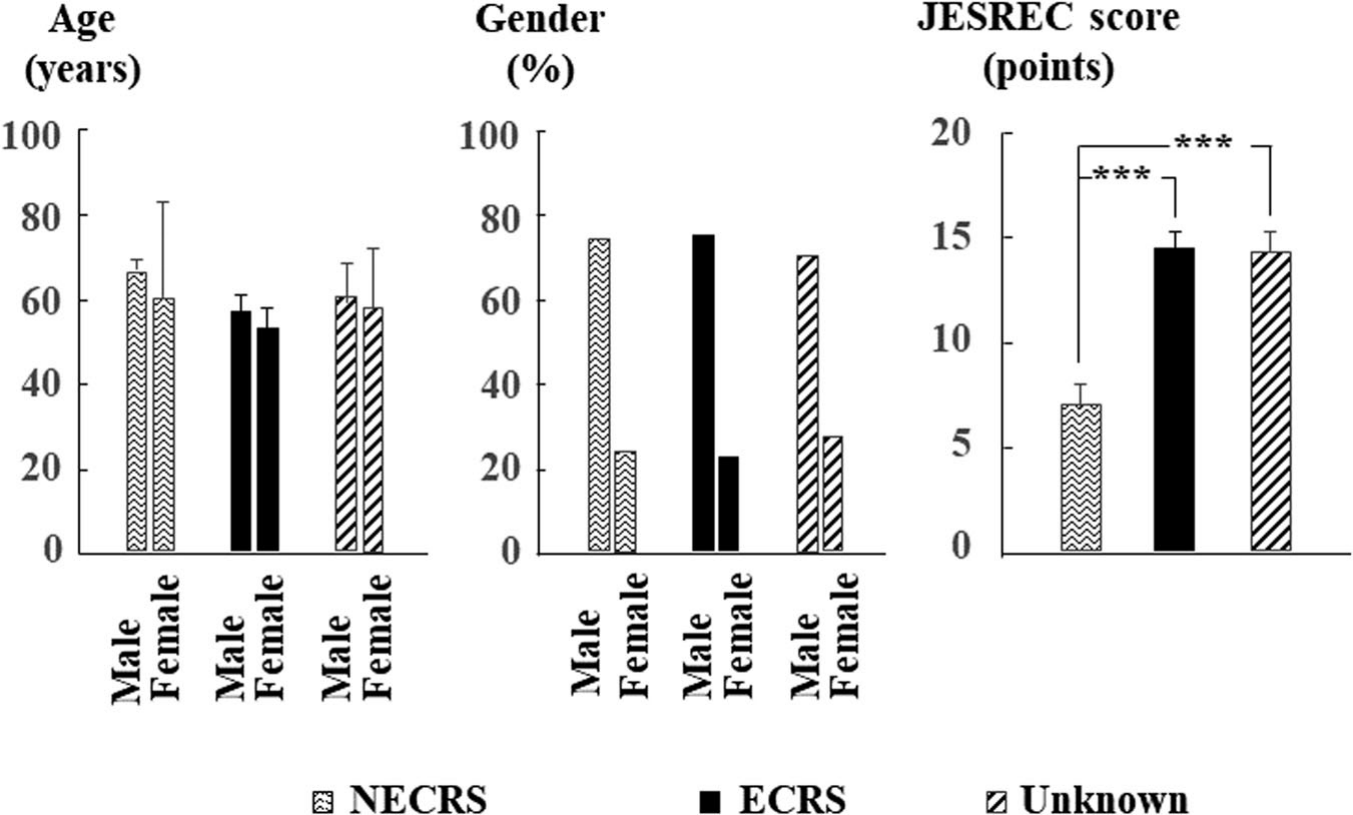
Subject characteristics. Bar graphs show mean ages, sex ratios (male: female), and JESREC scores among NECRS, ECRS, and unknown groups. ***P < .001. ECRS, eosinophilic chronic rhinosinusitis; JESREC, Japanese Epidemiological Survey of Refractory Eosinophilic Chronic Rhinosinusitis; NECRS, non‐ECRS

As shown in Figure 1, no significant differences were observed in mean ages of patients in ECRS, NECRS, and unknown groups. However, the ratio of male patients in all three groups was obviously higher than that of female patients. Compare with the NECRS group, ECRS and unknown groups had significantly higher JESREC scores.

**Figure 2:**
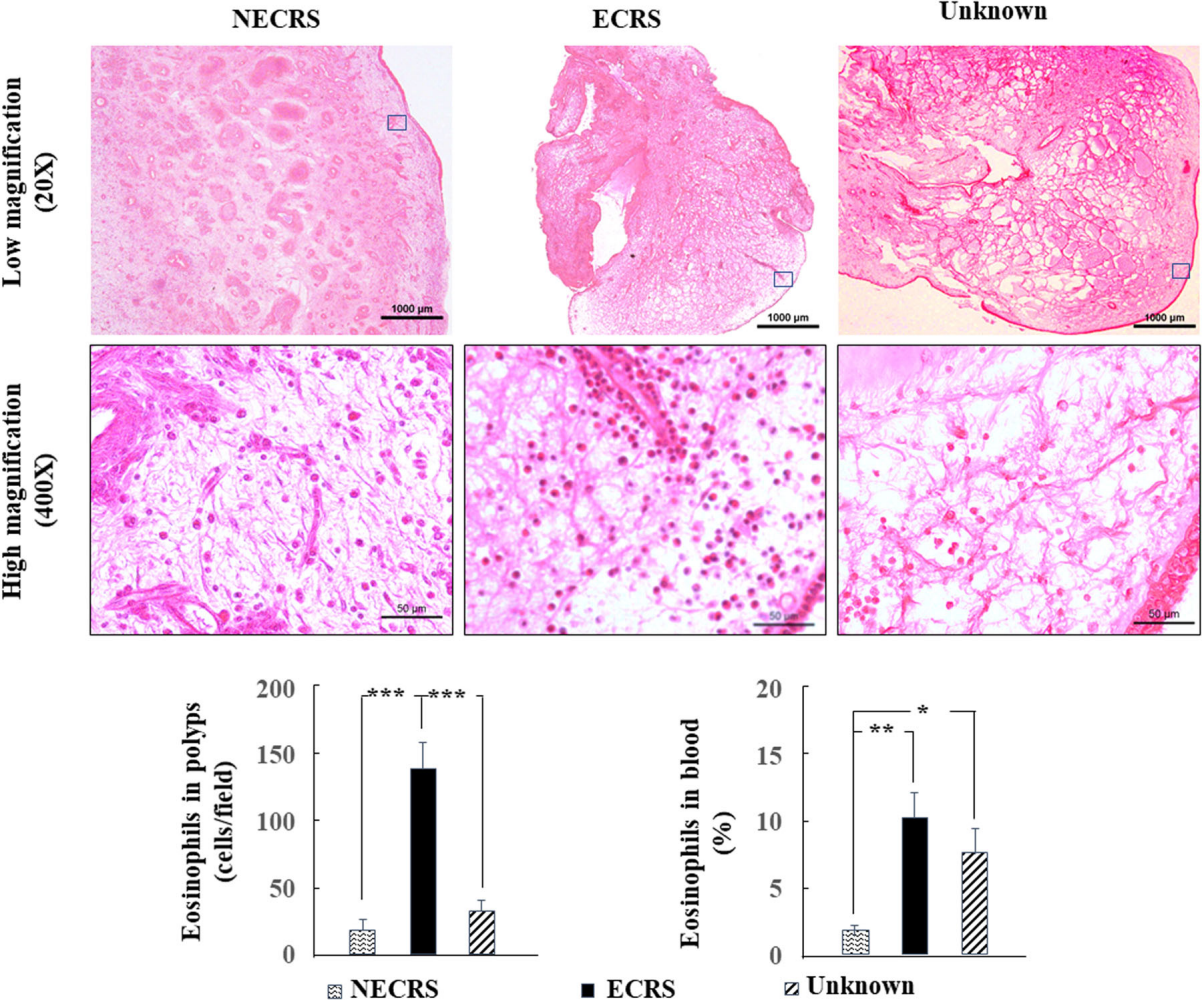
Changes in eosinophils in polyp tissues and blood. Representative hematoxylin and eosin‐stained images and quantitation of eosinophil numbers in nasal polyp tissues and blood are shown. *P < .05, **P < .01, ***P < .001. ECRS, eosinophilic chronic rhinosinusitis; NECRS, non‐ECRS

Figure 2 shows representative HE staining for resected nasal polyps. Eosinophil infiltration in resected nasal polyps was most evident in the ECRS group. The ratio of blood eosinophils in the ECRS group was also significantly higher than that of the NECRS group (*P *<* *.01). Although the number of infiltrating eosinophils in the unknown group did not differ from that of the NECRS group, the ratio of blood eosinophils in the unknown group was significantly higher than that of the ECRS group (*P *<* *.05).

**Figure 3:**
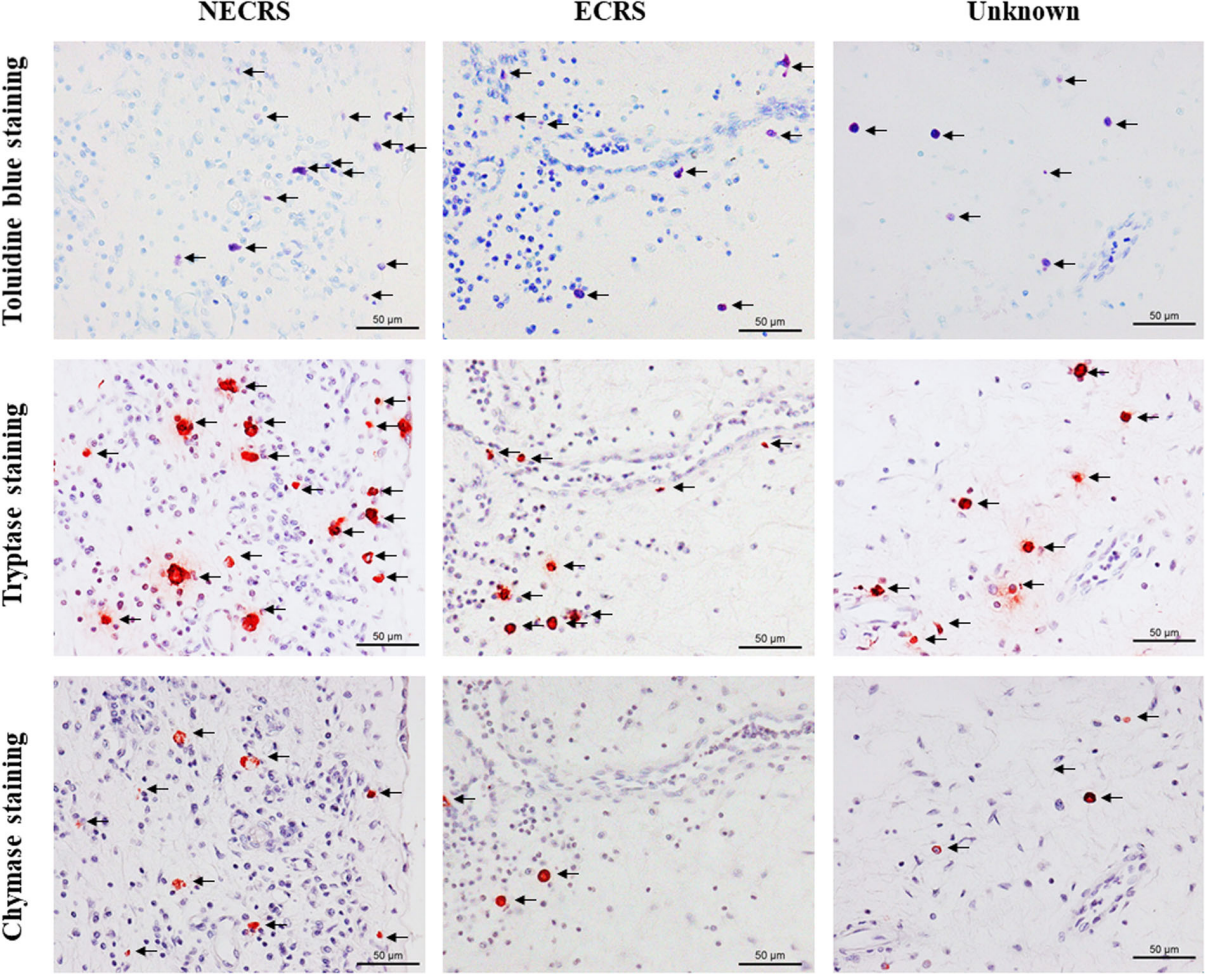
Mast cell expression patterns of NECRS, ECRS, and unknown groups. Representative images of toluidine blue staining and immunohistochemical staining for tryptase and chymase for each group are shown. ECRS, eosinophilic chronic rhinosinusitis; NECRS, non‐ECRS

Figure 3 shows representative toluidine blue staining and immunohistochemical staining for tryptase and chymase on serial polyp cross sections from the three groups. Compared with images from ECRS and unknown patients, a large number of mast cells and tryptase‐ and chymase‐positive cells were observed in sections from NECRS patients.

**Figure 4:**
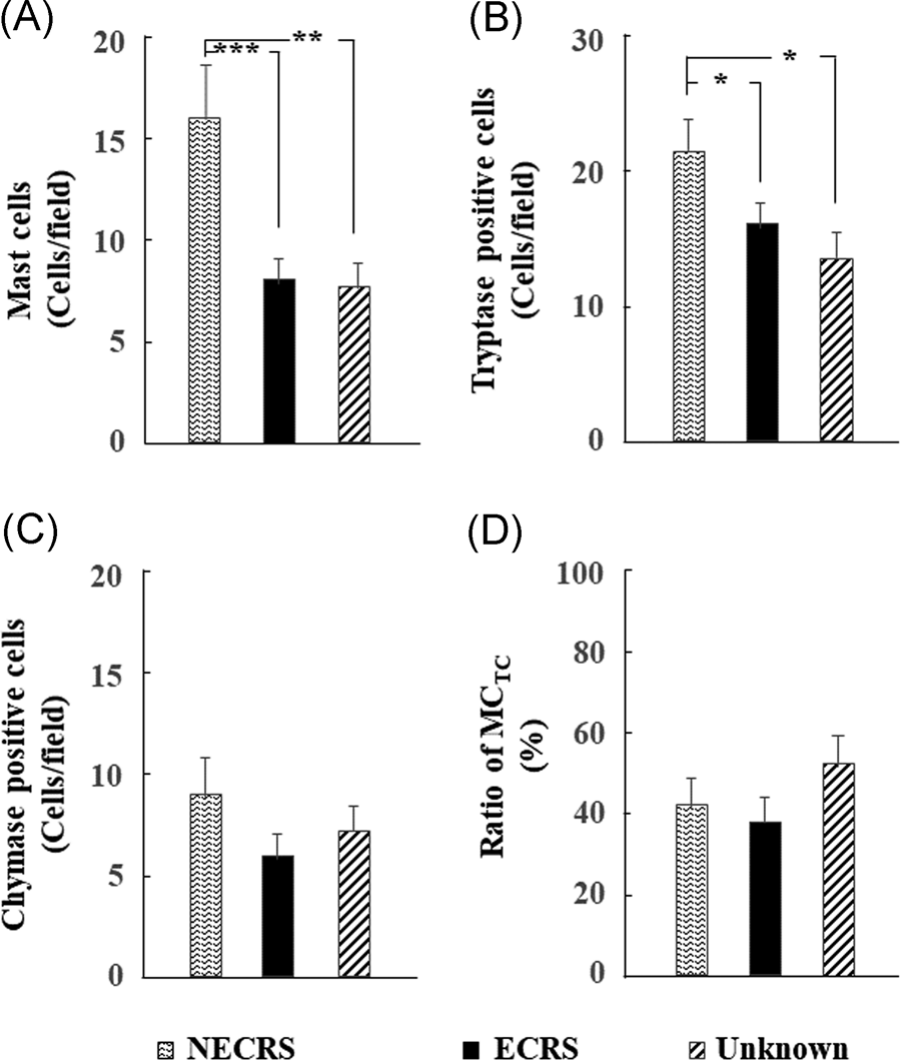
Quantitative analysis of mast cells and protease‐positive cells in resected polyp tissues from NECRS, ECRS, and unknown groups. *P < .05, **P < .01, ***P < .001. ECRS, eosinophilic chronic rhinosinusitis; NECRS, non‐ECRS

We next sought to determine protein expression profiles of mast cells in nasal polyps from CRSwNP patients with different phenotypes. The numbers of mast cells and tryptase‐positive cells were significantly lower in ECRS and unknown groups (Figure 4A,B; *P *<* *.001, ECRS vs NECRS; *P *<* *.01, unknown vs NECRS, for mast cells; *P *<* *.05, ECRS vs NECRS; *P *<* *.05, unknown vs NECRS, for tryptase‐positive cells). However, the number of chymase‐positive cells in polyp cross sections did not significantly differ among the three groups (Figure 4C). As mentioned in Section 2, two distinct subtypes of human mast cells have been described based on protease content, namely, MC_TC_ and MC_T_. Although the total number of mast cells evaluated with toluidine blue staining and tryptase immunostaining was significantly lower in ECRS and unknown groups than the NECRS group, the MC_TC_ ratio was not significantly different among the three groups (Figure 4D).

**Figure 5:**
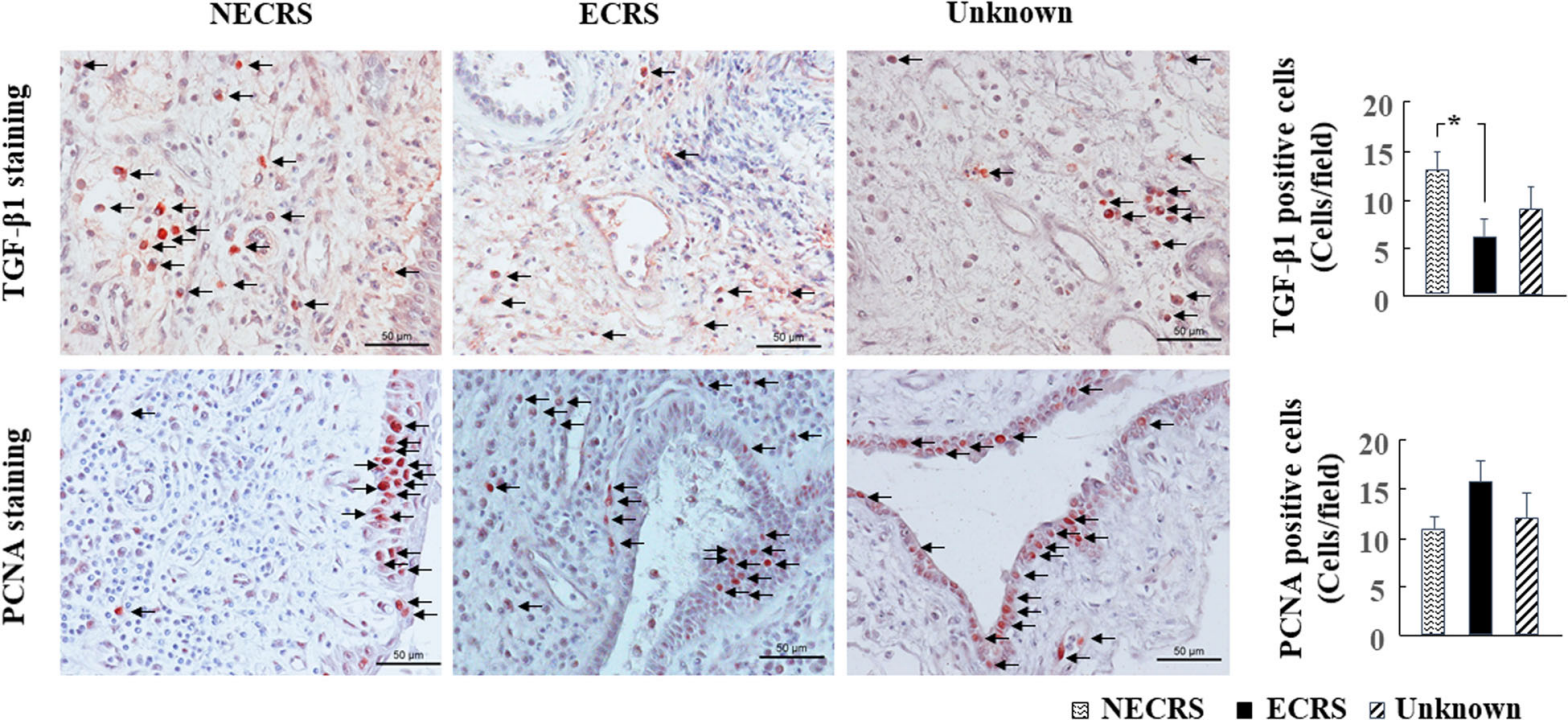
Immunohistochemical staining for TGF‐β1 and PCNA. Representative images of TGF‐β1 and PCNA staining in nasal polyp tissues of NECRS, ECRS, and unknown groups are shown. *P < .05. ECRS, eosinophilic chronic rhinosinusitis; NECRS, non‐ECRS; PCNA, proliferating cell nuclear antigen; TGF‐β1, transforming growth factor β1

Compared with the NECRS group, the number of TGF‐β1‐positive cells was significantly lower in the ECRS group (Figure 5; *P *<* *.05). However, no significant difference in the number of TGF‐β1‐positive cells was found between NECRS and unknown groups. PCNA is a nuclear protein that regulates cell growth by promoting DNA replication.^13^ In the present study, we found that epithelial cells actively proliferated in resected polyp tissues of all three groups. In addition, the number of PCNA‐positive cells tended to increase in the ECRS group (Figure 5).

**Figure 6:**
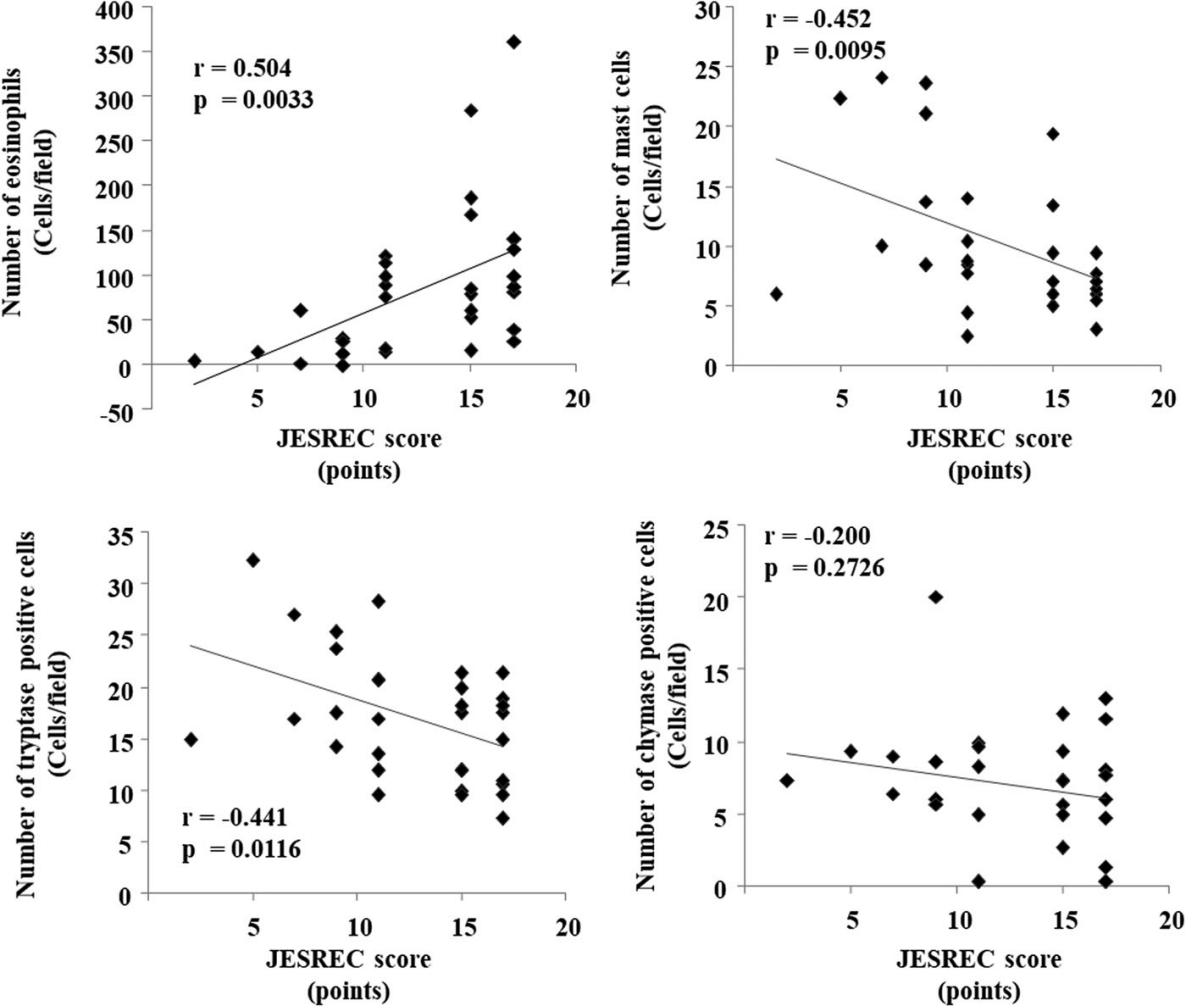
Linear regression analyses. Correlations between the JESREC score and number of eosinophils, mast cells, tryptase‐positive cells, and chymase‐positive cells are shown. JESREC, Japanese Epidemiological Survey of Refractory Eosinophilic Chronic Rhinosinusitis

A significant positive correlation was detected between the JESREC score and number of eosinophils (*r *=* *0.504, *P *<* *.01). In contrast, the numbers of mast cells and tryptase‐positive cells were negatively correlated with the JESREC score in all included samples (n = 32, Figure 6; mast cells, *r* = 0.452, *P *<* *.01; tryptase‐positive cells, *r* = 0.441, *P *<* *.05). No significant correlation was found between the JESREC score and number of chymase‐positive cells.

**Figure 7:**
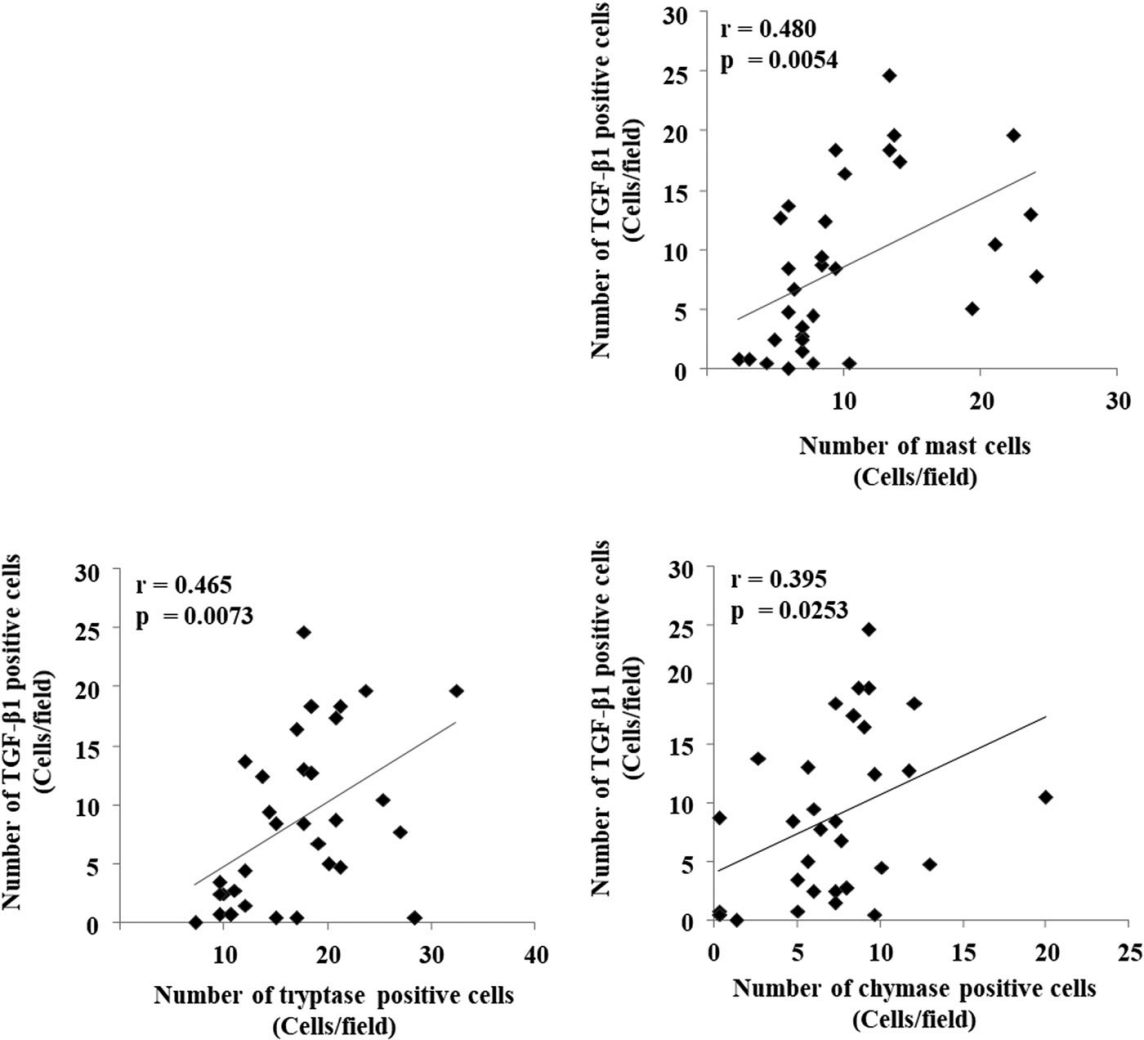
Linear regression analyses. Correlations between the number of TGF‐β1‐positive cells and the number of mast cells, tryptase‐positive cells, and chymase‐positive cells are shown. TGF‐β1, transforming growth factor β1

Significant positive correlations were detected between the number of TGF‐β1‐positive cells and the number of mast cells, tryptase‐positive cells, and chymase‐positive cells (Figure 7; mast cells, *r* = 0.48, *P *<* *.01; tryptase‐positive cells, *r* = 0.465, *P *<* *.01; *r* = 0.395, *P *<* *.05).

**Figure 8:**
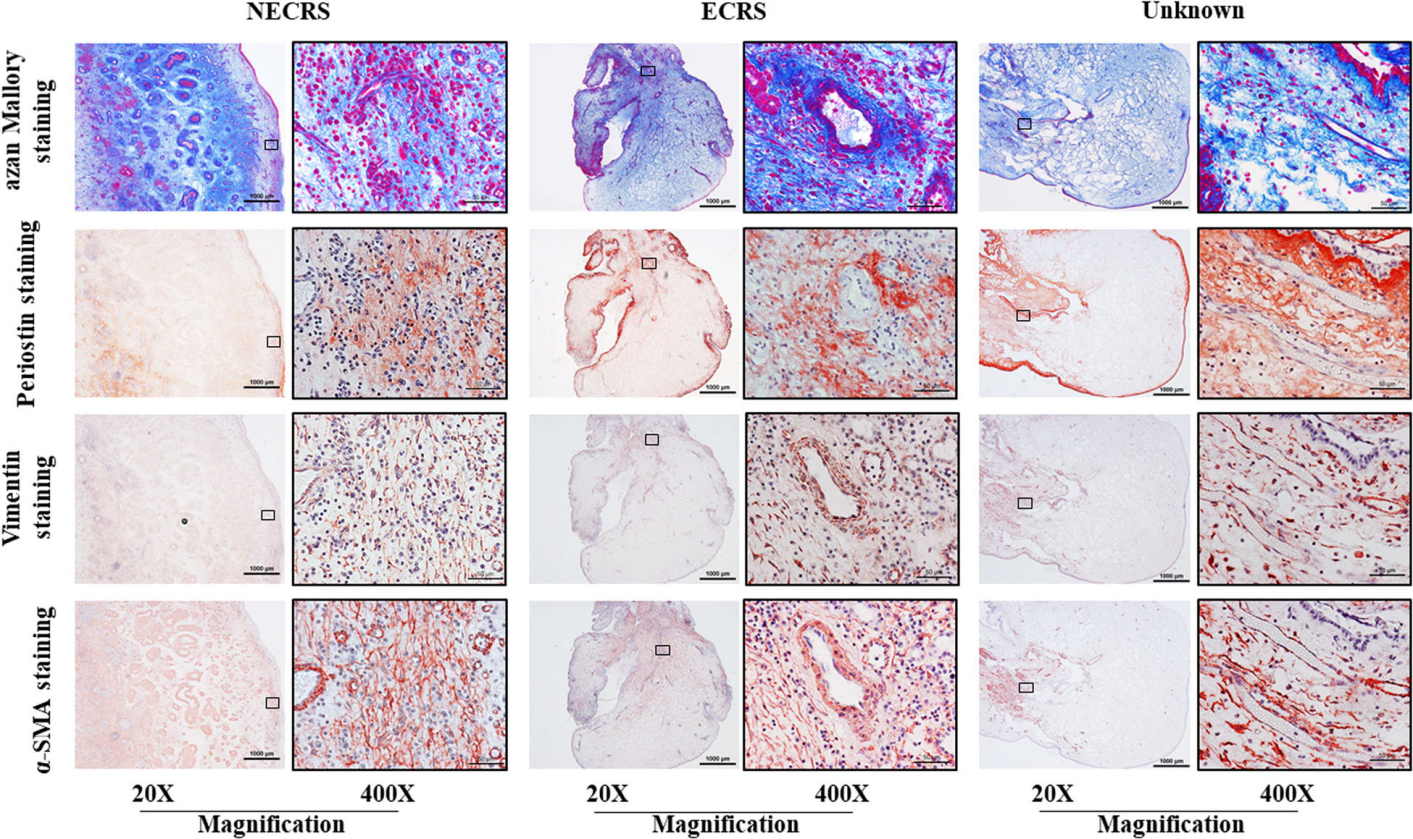
Identification of cellular and extracellular components. Azan Mallory staining was used to detect collagen deposition in the extracellular matrix. Distribution of periostin, a secreted extracellular matrix protein, was also examined by immunohistochemistry. Vimentin and α‐SMA staining was performed on serial cross sections to identify fibroblasts and myofibroblasts, respectively. ECRS, eosinophilic chronic rhinosinusitis; NECRS, non‐ECRS; α‐SMA, alpha‐smooth muscle actin

Azan Mallory staining, collagen as an extracellular matrix was widely distributed among all cross sections from the three groups. Periostin was heterogeneously distributed among these cross sections, with positive staining most evident at the lateral margin of polyps (Figure 8), although periostin‐positive staining was also occasionally found in the interstitium of polyp parenchymal tissues. Vimentin is a structural protein that is mainly expressed in mesenchymal cells, such as fibroblasts and myofibroblasts, as well as in endothelial cells.^14^ α‐SMA as a contractile protein is mainly expressed in contractile vascular smooth muscle cells . On the other hand, it is also expressed during the time when the phenotypic change from fibroblast to myofibroblast occurs.^15^ Therefore, the proportions of fibroblasts and myofibroblasts can be calculated by the determination of vimentin and α‐SMA expression on serial sections from polyp tissues. As shown in Figure 8, spindle‐shaped vimentin‐positive cells were widely dispersed throughout polyp tissues from all three groups. Most of this staining localized with α‐SMA‐positive staining, indicating that the majority of the cellular component of mesenchymal cells is myofibroblasts in the three groups (Figure 8). Because the main objective of the present study was to analyze mast cell expression patterns in CRSwNP patients, we did not perform quantitative analysis of extracellular matrix, fibroblasts, and myofibroblasts.

## 4 DISCUSSION

The postoperative management and support of ECRS patients in Japan have substantially improved following the proposal of diagnostic criteria for ECRS in CRSwNP using the JESREC scoring system. Introduction of the incurable disease designation system has given definitively diagnosed ECRS patients a great benefit not only in the availability of postoperative maintenance therapies but also in decreased medical treatment costs. However, some CRSwNP patients are still missed by this diagnostic system mainly because they present with too few eosinophils in polyp tissues. The sensitivity and specificity for the prediction of postoperative polyp recurrence in CRSwNP patients with JESREC scores >11 are 83% and 66%, respectively.^5^ Therefore, there remains a high risk for polyp recurrence in some undetected ECRS patients.

In the present study, a significant positive correlation was detected between the JESREC score and number of eosinophils in resected polyps, supporting high polyp recurrence in ECRS patients.^16^ In contrast, the numbers of mast cells and tryptase‐positive cells were negatively correlated with the JESREC score in all included subjects, indicating that a decrease in the number of mast cells in resected polyps of CRSwNP patients may reflect polyp recurrence to some extent. Human mast cells are usually classified as MC_TC_ or MC_T_ according to the amount of tryptase and chymase stored in secretory granules.^10^ In the present study, we found that the total number of mast cells evaluated with toluidine blue staining and tryptase immunostaining was significantly lower in ECRS and unknown patients than NECRS patients. Conversely, MC_TC_ and MC_T_ ratios did not significantly differ among the three groups, indicating that only the total number of mast cells decreased while MC_TC_ and MC_T_ ratios remained unchanged.

In the present study, although the detailed relationship between polyp recurrence and mast cell downregulation is unknown, the decrease in TGF‐β1 expression may be partly related to this pathophysiology. As shown in Figure 5, the number of TGF‐β1‐positive cells was significantly lower in ECRS patients compared with NECRS patients. We also found that the number of TGF‐β1‐positive cells was positively correlated with the number of mast cells (Figure 7). Therefore, the low TGF‐β1 expression in ECRS patients may be partly related to the decrease in the number of mast cell number in polyps. TGF‐β1 is secreted from many different cell types including mast cells.^11^, ^17^ Secreted TGF‐β1 protein participates in the regulation of cell growth, cell proliferation, cell differentiation, and apoptosis.^18^, ^19^ However, these regulatory effects of TGF‐β1 are sometimes biphasic, and TGF‐β1 can exert opposing effects depending on local concentration. For example, Cordeiro et al reported that optimal concentrations for fibroblast proliferation ranged from 9‐10 to 10‐12 M , while concentrations above or below this range suppressed fibroblast growth. The discrepancy in TGF‐β1 expression in CRSwNP patients was also reported. Some researchers reported greater TGF‐β1 expression in CRSwNP patients compared with healthy subjects,^20^, ^21^ while others found the opposite.^22^, ^23^ Although we did not analyze TGF‐β1 expression in the nasal mucosa from healthy subjects, our present finding of TGF‐β1 expression may be consistent with the latter result.

The pathophysiological mechanism of polyp formation under CRS has been extensively explored. The relationship between periostin and nasal polyp formation has received great attention.^9^, ^24^ For example, the periostin expression level in polyp tissues and plasma is associated with the severity of CRSwNP, and serum periostin level has been recommended as a novel biomarker for postoperative recurrence of CRSwNP. In the present study, we found that periostin was heterogeneously distributed among polyp cross sections and similar staining patterns were observed among the three groups. As shown in Figure 8, periostin expression was most evident at the lateral margin of polyps, which supports the previous finding that periostin in nasal tissues is mainly produced by epithelial cells and deposited into subepithelial regions.^25^ Periostin expression was also occasionally found in the interstitium of polyp parenchymal tissues, which are rich in myofibroblasts (Figure 8), indicating myofibroblasts and fibroblasts also secrete periostin.^26^

We could not determine the optimal cutoff number of mast cells to predict polyp recurrence after surgery in CRSwNP because follow‐up of these patients is currently ongoing. Another limitation of determining the optimal cutoff value is the small number of recruited subjects. Therefore, a multi‐center study with larger sample size and longer follow‐up of suspected ECRS patients, especially those classified as unknown CRSwNP patients, may be necessary.

In conclusion, the present study indicated that the number of mast cells in resected polyps of ECRS patients was significantly lower than in those from NECRS patients. Moreover, since a significant negative correlation between the JESREC score and the number of mast cells was detected, enumeration of mast cells in resected polyps may be another approach to predict postoperative polyp recurrence in CRSwNP patients.

## CONFLICT OF INTERESTS

The authors declare that there is no conflict of interests.

## AUTHOR CONTRIBUTIONS

DJ and YT conceived and managed the study and wrote the manuscript. ST and KS collected polyp samples and performed histological staining. ST critically revised the manuscript.

## DATA ACCESSIBILITY

The data that support the findings of this study are available on request from the corresponding author, Denan Jin (E‐mail: pha012 @osaka‐med.ac.jp).

## ETHICS STATEMENT

Informed consent was obtained from all patients for this study. This experimental protocol was also approved by the Human Studies Committee of the Graduate School of Medicine and Faculty of Medicine, Osaka City University (authorization number: 3606).
